# Hepatocyte-specific loss of melanocortin 1 receptor disturbs fatty acid metabolism and promotes adipocyte hypertrophy

**DOI:** 10.1038/s41366-024-01600-9

**Published:** 2024-08-08

**Authors:** Keshav Thapa, Bishwa Ghimire, Kisun Pokharel, Minying Cai, Eriika Savontaus, Petteri Rinne

**Affiliations:** 1 Research Centre for Integrative Physiology and Pharmacology, Institute of Biomedicine, University of Turku, Turku, Finland; 2 Drug Research Doctoral Programme (DRDP), University of Turku, Turku, Finland; 3 Institute for Molecular Medicine Finland (FIMM), HiLIFE Helsinki Institute of Life Science, University of Helsinki, Helsinki, Finland; 4 Medicity Research Laboratory, University of Turku, Turku, Finland; 5 Natural Resources Institute Finland (Luke), Jokioinen, Finland; 6 Department of Chemistry and Biochemistry, University of Arizona, Tucson, AZ USA; 7 Turku Center for Disease Modeling, University of Turku, Turku, Finland; 8 Unit of Clinical Pharmacology, Turku University Hospital, Turku, Finland

**Keywords:** Fat metabolism, Obesity

## Abstract

**Background/objectives:**

Melanocortins mediate their biological functions via five different melanocortin receptors (MC1R - MC5R). MC1R is expressed in the skin and leukocytes, where it regulates skin pigmentation and inflammatory responses. MC1R is also present in the liver and white adipose tissue, but its functional role in these tissues is unclear. This study aimed at determining the regulatory role of MC1R in fatty acid metabolism.

**Methods:**

Male recessive yellow (Mc1r^e/e^) mice, a model of global MC1R deficiency, and male hepatocyte-specific MC1R deficient mice (Mc1r LKO) were fed a chow or Western diet for 12 weeks. The mouse models were characterized for body weight and composition, liver adiposity, adipose tissue mass and morphology, glucose metabolism and lipid metabolism. Furthermore, qPCR and RNA sequencing analyses were used to investigate gene expression profiles in the liver and adipose tissue. HepG2 cells and primary mouse hepatocytes were used to study the effects of pharmacological MC1R activation.

**Results:**

Chow- and Western diet-fed Mc1r^e/e^ showed increased liver weight, white adipose tissue mass and plasma triglyceride (TG) concentration compared to wild type mice. This phenotype occurred without significant changes in food intake, body weight, physical activity or glucose metabolism. Mc1r LKO mice displayed a similar phenotype characterized by larger fat depots, increased adipocyte hypertrophy and enhanced accumulation of TG in the liver and plasma. In terms of gene expression, markers of de novo lipogenesis, inflammation and apoptosis were upregulated in the liver of Mc1r LKO mice, while enzymes regulating lipolysis were downregulated in white adipose tissue of these mice. In cultured hepatocytes, selective activation of MC1R reduced ChREBP expression, which is a central transcription factor for lipogenesis.

**Conclusions:**

Hepatocyte-specific loss of MC1R disturbs fatty acid metabolism in the liver and leads to an obesity phenotype characterized by enhanced adipocyte hypertrophy and TG accumulation in the liver and circulation.

## Introduction

Obesity has become a major health concern worldwide and is highly associated with comorbidities such as type 2 diabetes, dyslipidemia, non-alcoholic fatty liver disease (NAFLD), and cardiovascular disease [1, 2]. NAFLD is characterized by an increase in intrahepatic triglyceride (TG) content, which occurs when the rate of fatty acid (FA) input exceeds the rate of FA output. Therefore, the accumulation of TGs in the liver is determined by a complex interaction between hepatic FA uptake, de novo lipogenesis (DNL) and fatty acid oxidation (FAO). In the setting of NAFLD, majority of hepatic FAs originate from adipose tissue due the development of insulin resistance and enhanced lipolysis [3, 4]. Furthermore, increased hepatic DNL contributes to the accumulation of TGs in the liver of NAFLD patients. The liver and adipose tissue have thus a central role in the regulation of whole-body energy homeostasis, but in obesity-associated NAFLD, persistent lipid overload triggers metabolic stress and inflammation, and disturbs the cross-talk between these tissues [5].

The melanocortins are a group of small peptide hormones derived from post-translational processing of proopiomelanocortin precursor protein and consist of adrenocorticotropic hormone and melanocyte-stimulating hormones (α-, β-, and γ-MSH) [6]. Melanocortins mediate their biological actions via G-protein-coupled melanocortin receptors (MC1R - MC5R) that are widely expressed in the central nervous system and in peripheral tissues. Melanocortin receptors regulate a wide array of physiological functions including melanogenesis, steroidogenesis, exocrine secretion, sexual function, inflammation, and energy homeostasis [7, 8]. MC1R possesses high affinity for α-MSH and is known for its role in skin pigmentation [9]. MC1R is also abundantly expressed in leukocytes, where it mediates anti-inflammatory actions [10]. Interestingly, earlier studies have found that MC1R is present in human and mouse adipocytes, and upregulated in the adipose tissue of subjects with obesity [11–14]. Intriguingly, α-MSH and other melanocortin peptides induce lipolysis in white adipocytes, but this effect has been mostly linked to MC5R activation [15, 16]. However, in human adipocytes or adipose tissue explants, MC1R activation does not induce a regulatory effect on lipolysis [17]. In contrast, MC1R stimulation with α-MSH inhibits cell proliferation in human preadipocytes [17]. Consequently, the functional role of MC1R in adipocytes remains unknown. It is also unclear whether the presence of MC1R in other tissues could have an impact on lipid metabolism.

Here, we aimed to study whether global MC1R deficiency renders mice susceptible for enhanced fat accumulation and obesity. We observed that mice with global MC1R deficiency had normal body weight but significantly increased liver weight and adiposity. Gene expression analyses suggested enhanced de novo lipogenesis in the liver of these mice, while no consistent transcriptional changes were observed in the adipose tissue. Thus, we hypothesized that the obesity phenotype is driven by MC1R deficiency in hepatocytes. Indeed, mice with hepatocyte-specific loss of MC1R recapitulated the obesity phenotype as evidenced by increased white adipose tissue mass and liver weight, and elevated triglyceride accumulation in the liver and circulation. This study demonstrates, for the first time, the functional significance of MC1R in lipid metabolism, and that hepatocytes appear to be the responsible cells for mediating this regulatory function of MC1R.

## Materials and methods

### Mouse models and experimental design

All experimental procedures were performed on adult (3–6 months) male mice. Mice were maintained on a fixed 12-hour light/dark cycle (lights on at 7 am and off at 7 pm), temperature (21 ± 1 °C) and humidity (50 ± 5%) at Central Animal laboratory, University of Turku. Mice were housed in individually-ventilated cages with a bedding of wood shavings and suitable nesting material, and the cages were changed to clean ones every week. Food and tap water were available *ad libitum*. The number of mice included in each experiment are mentioned in the figure legends. Mice were allocated to receive either a regular chow diet (# 2916 C, Teklad Global diet, Envigo) or Western-type diet (RD Western diet, D12079B, Research diets Inc, NJ, USA) for the entire experimental duration using simple randomization. In each experiment, mice were euthanized *via* CO_2_ asphyxiation after a four-hour fast and blood was withdrawn by cardiac puncture. White adipose tissue (WAT) depots and whole liver were excised, weighted, and rapidly dissected and frozen until further analysis. All animal procedures used in our studies were approved by the national Animal Experiment Board in Finland (License Numbers: ESAVI/6280/04.10.07.2016 and ESAVI/1260/2020) and conducted in accordance with the institutional and national guidelines for the care and use of laboratory animals.

Recessive yellow mice (Mc1r^e/e^), that are homozygous for the Mc1r^e^ allele and lack functional MC1R due to a single base deletion mutation, and their non-mutant littermate controls (WT) were obtained from the Jackson Laboratory (Strain # 000060, Bar Harbor, ME, USA). Mc1r^e/e^ and WT mice were kept on a regular chow diet or Western-type diet for 12 weeks starting at eight weeks of age. Mc1r^e/e^ and WT mice were housed in groups of littermates (2–5 mice/cage) for the entire experiment. Additionally, hepatocyte-specific MC1R knockout mice (Mc1r LKO) were generated by breeding mice homozygous for a floxed *Mc1r* allele (*Mc1r*^*fl/fl*^) mice (the Jackson Laboratory, strain #029239) [18] with transgenic *Alb*^*Cre/+*^ mice (B6N.Cg-Speer6-ps1Tg(Alb-cre)1MGn/J, the Jackson Laboratory, strain #018961). These genetically modified mice were individually identified with earmarks and housed with littermates with same sex (1–6 mice/cage). Age-matched *Mc1r*^*fl/fl*^ mice were used as controls. Eight-week-old control and Mc1r LKO mice were placed on a Western-type diet for 12 weeks. Efficient recombination of the loxP-flanked allele and downregulation of *Mc1r* in the liver of Mc1r LKO mice have been previously demonstrated [19].

### Cell culture and primary mouse hepatocyte isolation

HepG2 cells were used as an in vitro model to investigate the effects of MC1R activation as previously described [19]. Briefly, HepG2 cell line (authenticated by STR profiling, American Type Culture Collection, Rockville, MD, USA) was maintained in complete growth medium DMEM (Dulbecco’s modified Eagle’s medium; Sigma-Aldrich) supplemented with 10% (v/v) heat-inactivated fetal bovine serum (FBS; Thermo Fisher Scientific, Gibco, MA, USA), 100 U/ml penicillin (Gibco), 100 µg/ml streptomycin (Gibco) at 37 °C in a humid atmosphere with 5% CO_2_. For experiments, cells were seeded on 12- or 24-well plates and treated with α-MSH (abcam, # ab120189) or the selective MC1R agonist LD211 (compound 28 in the original publication) [20], as indicated in the figure legends.

To isolate primary hepatocytes, 4-month-old male C57B1/6 N mice were anesthetized using a cocktail of ketamine (Vetmedic) and medetomidine (MSD Animal Health). After a midline incision, liver, portal vein, and inferior vena cava were exposed to insert a 24 G catheter into the inferior vena cava. Thereafter, the portal vein was cut open and the liver was perfused with pre-warmed liver perfusion medium (Gibco, #17701-038) at a rate of 4.5 ml/min for 3–5 min until the flushed-out liquid was clear. The peristaltic pump was then stopped, and the perfusion medium was changed to liver digest medium containing high glucose DMEM, 1% Pen/Strep, 10 µg/ml HEPES, 10% FBS and 1 mg/ml collagenase IV. Pumping was continued until 30 ml of the medium was used, and then the liver was excised and placed on 100 mm petri dish containing 20 ml of cold wash medium (Gibco, # 17704024) on ice. Gall bladder was removed, and liver was teared down with the forceps. Cells were poured through a 70 µm filter into a 50 ml falcon tube and centrifuged (50 × *g*, 3 min, +4 °C). Cell pellet was resuspended in 10 ml of cold wash medium and mixed thoroughly with 10 ml of cold 90% Percoll-PBS solution (9 ml of Percoll, Sigma P1644, 1 ml of 10X PBS) solution. Cell suspension was centrifuged at 300 × *g* for 10 min at 4 °C, the pellet was washed with 20 ml of cold wash medium and cell suspension was centrifuged again (50 × *g*, 3 min, +4 °C). Finally, cell pellet was resuspended in 20 ml of maintenance medium (William’s E medium, GlutaMAX Supplement, Gibco, # 32551087) supplemented with 10% FBS and 1% Pen/ Strep. Cells were seeded into 12-well (2 × 10^5^ cells/well) or 6-well plates (4 × 10^5^ cells/well), incubated at 37 °C in a humid atmosphere with 5% CO_2_, and used in experiments after around 12 h from the plating.

### Metabolic studies

Body weight was measured weekly during the 12-week diet intervention (from 8 to 20 weeks of age). Food was changed and measured on a weekly basis. Food intake was measured from group-housed mice by subtracting remaining food, including spilled food in cages, from a weighed weekly aliquot of food. Energy intake was calculated based on 19.54 kJ/g (4.67 kcal/g) for the Western diet and 15.99 kJ/g (3.82 kcal/g) for the chow diet (caloric values obtained from Research Diets). Body composition was determined prior to the initiation of the diet intervention and at the end of the 12-week monitoring period using quantitative nuclear magnetic resonance (NMR) scanning (EchoMRI-700, Echo Medical Systems, Houston, TX, USA). At sacrifice, whole liver was excised, and its composition (total amount of fat, free water, and lean mass) was measured with an EchoMRI-700 device. Glucose tolerance test (GTT) was performed at the end of the experiment for Mc1r^e/e^ mice and twice for Mc1r LKO mice during the 12-week diet intervention. The first GTT was performed at week 4 from the start of diet intervention and the last GTT was performed at week 11 just before the end of the experiment. Mice were fasted for 4 hours before performing GTT. A basal glucose reading was obtained, and mice were then injected intraperitoneally with glucose at dose of 2.5 g/kg lean mass. Blood samples were withdrawn via tail vein and blood glucose levels were measured at 20-, 40-, 60-, and 90-min time points post-injection.

### Spontaneous locomotor activity

Spontaneous locomotor activity of individually-housed WT and Mc1r^e/e^ mice was monitored with a photo-beam recording system (San Diego Instruments, San Diego, CA, USA) at the end of the diet intervention. After 24-h habituation, locomotor activity was measured over 10 min intervals for 24 h.

### RNA isolation, cDNA synthesis and quantitative RT-PCR

HepG2 cells and primary mouse hepatocytes were collected into QIAzol Lysis Reagent and total RNA was extracted using Direct-zol RNA Miniprep (Zymo Research, CA, USA). Liver and gonadal white adipose tissue (gWAT) samples were homogenized in QIAzol Lysis Reagent (Qiagen, Venlo, Netherlands) with metal beads (Qiagen) using the Qiagen TissueLyser LT Bead Mill (Qiagen). Thereafter, total RNA from each sample was extracted and reverse-transcribed to cDNA with PrimeScript RT reagent kit (Takara Clontech) following the manufacturer’s instructions. The quantity and purity of RNA were measured by Nanodrop (ThermoFisher Scientific). Quantitative real-time polymerase chain reaction (RT-qPCR) was performed using SYBR Green protocols (Kapa Biosystems, MA, USA), as previously described [21, 22]. Target gene expression was normalized to the geometric mean of two housekeeping genes (*Ppia* and *Mrps18a* for mouse genes, *GAPDH* and *RPS18* for human genes) using the delta-Ct method and results are presented as relative transcript levels (2^-ΔΔCt^). Primers sequences are presented in Tables S1, S2.

### Hepatic transcriptomic analyses

RNA sequencing (RNA-Seq) analyses were performed on highly-pure RNA samples from the liver of Western diet-fed control (*n* = 4) and Mc1r LKO (*n* = 4) mice at the Finnish Functional Genomics Center according to the manufacturer’s protocols (Illumina, Cambridge, UK). The binary base call (BCL) sequence files generated by Illumina Novaseq 6000 were converted to FASTQ files using Illumina’s bcl2fastq (version 2.18.0.12). The FASTQ files were processed using FIMM-RNAseq (version 3.0.0) data analysis pipeline [23]. The T overhang added by the library preparation workflow was removed from forward and reverse reads. Trim-galore (version 0.6.6) was used for quality trimming and adapter removal [24]. STAR (version 2.7.6a) was used for read alignment and Subread (version 2.0.1) was used for gene quantification [25]. Clean RNA-Seq reads were aligned to the mouse reference genome GRCh38 (release) from Ensembl. Low gene counts were filtered using edgeR’s [26] (version 3.36.0) *filterByExpr* function utilizing default parameters and normalized by TMM (trimmed mean of M-values) normalization method. Differential gene expression analysis was carried out using DGA-quik (https://version.helsinki.fi/fimm/dga-quik) written in R programming language (https://www.r-project.org). The gene expression profile between control and Mc1r LKO mice were compared. A total of 635 differentially expressed genes (DEGs) were yielded from the analysis after filtering with *p*-value cutoff < 0.05 and absolute log fold-change (logFC) ≥ 0.25. The DEGs were further tested for their over-representation in Gene Ontology (GO) terms using topGO [27] (version 2.46.0), a Bioconductor R package. The over-represented GO terms produced by the analysis were further filtered based on a cutoff *p.adj* ≤ 0.05.

### Histological analysis

Liver and gonadal WAT were fixed with 10% buffered formalin solution for 24 h followed by dehydration in ethanol and embedding in paraffin. Blocks were cut into serial sections (4 µm-thick), which were stained with hematoxylin and eosin (H&E) and PicroSirius Red, as previously described [19]. ImageJ software (NIH, Bethesda, MD, USA) was used to measure adipocyte size and the extent of liver fibrosis after imaging the entire sections using an automated slide scanner (Pannoramic 250 or Pannoramic Midi, 3DHISTECH Kft., Budapest, Hungary).

### Plasma and liver lipid analyses

Terminal blood samples were withdrawn by cardiac puncture and plasma was obtained from EDTA-anticoagulated whole blood after centrifugation (380 *g*, 20 min, +4°C). Plasma total triglyceride concentrations were determined using enzymatic colorimetric assays (GPO-PAP, mtDiagnostics, Idstein, Germany) according to the manufacturer’s protocols. Non-esterified fatty acids (NEFA) concentration was determined using a commercially available kit (Free Fatty Acid Fluorometric Assay Kit, Item No. 700310, Cayman Chemicals, USA) following manufacturer’s instructions. Liver samples (~100 mg) were homogenized in 500 µl of phosphate-buffered saline (PBS) containing 0.1% NP-40 (Abcam) using Qiagen TissueLyser LT Bead Mill (QIAGEN, Venlo, Netherlands) and centrifuged to remove insoluble components [28, 29]. TG concentrations in the liver homogenates were quantified using GPO-PAP reagent.

### Protein extraction, SDS-PAGE and Western blotting

HepG2 cells and primary hepatocytes were lysed in ice-cold RIPA buffer (50 mM NaCl, 1% Triton X-100, 0,5% Sodium deoxycholate, 0,1% SDS, pH 8,0) supplemented with a cocktail of protease and phosphatase inhibitors (ThermoFisher, #A32961). For liver lysate preparation, liver samples were homogenized in ice-cold RIPA buffer with the addition of metal beads using Qiagen TissueLyser LT. Protein extracts were resolved by either 10% or 4–20% gradient (Bio-Rad) SDS-polyacrylamide gel electrophoresis (SDS-PAGE) and blotted onto either nitrocellulose membranes (GE Healthcare, Chalfont St. Giles, United Kingdom) or polyvinylidene difluoride (PVDF) membranes (Bio-Rad). Membranes were blocked with either 5% skimmed milk (Carl Roth) or 3% BSA (Tocris Bioscience) in 1X Tris-Buffered Saline (TBS) in 0.1% Tween^®^ 20 detergent (Fischer Bioreagents) for 1 h at room temperature (RT) and incubated with primary antibodies for ChREBP (Novus Biologicals, # NB400-135SS), caspase 3 (Abcam, # ab13847), BAX (Cell Signaling Tech, # 2772), phospho-JNK (Cell Signaling Tech, # 4668), JNK (R&D Systems, # AF-1387) at 4 °C overnight. Next day, membranes were washed and incubated with horseradish peroxidase (HRP)-conjugated secondary antibodies (Cell Signaling Tech) for 1 h at RT and proteins were detected using an enhanced chemiluminescence (ECL) kit (Millipore, MA, USA). Target protein expression was normalized to the expression of vinculin (Bio-Rad, #MCA465GA) to correct for loading, and signal strengths were analyzed using ImageJ software (NIH, Bethesda, MD, USA).

### Statistical analysis

All statistical analyses (except for RNA-Seq) were performed using GraphPad Prism 10 software (La Jolla, CA, USA). Sample sizes were empirically determined based on previous experience with diet-induced obesity models. Where possible, experiments were conducted and analyzed by blinded researchers. Statistical significance between the experimental groups was determined by two-tailed, unpaired Student’s *t* test or one-way or two-way ANOVA followed by Dunnett’s or Bonferroni post hoc tests. The D’Agostino and Pearson omnibus normality test method was utilized to check the normality of the data. Outliers in the data sets were identified using the regression and outlier removal (ROUT) method at Q-level of 1%. All data are presented as mean ± standard error of the mean (SEM). Results were considered significant for *p* values of less than 0.05.

## Results

### Global MC1R deficiency promotes white adipose tissue and liver weight without affecting body weight or composition

**Fig. 1:**
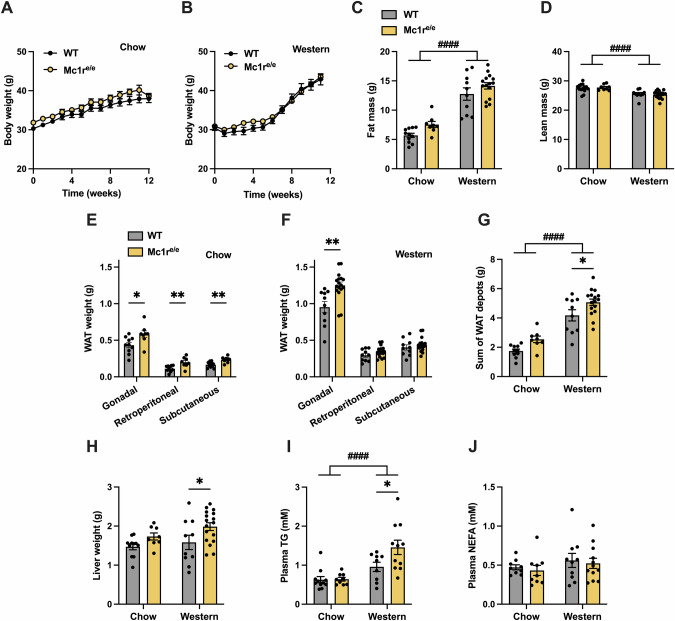
Mc1r^e/e^ mice with global MC1R deficiency show increased white adipose tissue mass and liver weight. **A**, **B** Body weight curves in chow- and Western diet-fed wild type (WT) and Mc1r^e/e^ mice. **C**, **D** Quantification of fat and lean mass by quantitative NMR scanning of whole-body composition in chow- and Western diet-fed WT and Mc1r^e/e^ mice at the end of the 12-week diet intervention. **E–G** White adipose tissue (WAT) weights and sum weights of WAT depots in chow- and Western diet-fed WT and Mc1r^e/e^ mice. **H** Liver weight in chow- and Western diet-fed WT and Mc1r^e/e^ mice. **I**, **J** Quantification of plasma triglyceride (TG) and non-esterified fatty acids (NEFA) concentrations in chow- and Western diet-fed WT and Mc1r^e/e^ mice. Values are mean ± SEM, *n* = 10–15 mice per group in each graph. *p < 0.05 and **p < 0.01 for the indicated comparisons by two-way ANOVA and Bonferroni *post hoc* tests. #### *p* < 0.0001 for the main effect of diet by 2-way ANOVA.WAT indicates white adipose tissue; TG triglyceride, NEFA non-esterified fatty acids.

To determine whether global MC1R deficiency promotes weight gain and adiposity, male WT and Mc1r^e/e^ mice were fed chow or Western diet for 12 weeks. Body weight was monitored weekly during the 12-week diet intervention period, but there were no significant differences between the genotypes in weight development in either of the diet groups (Fig. 1A, B). Likewise, body composition analysis by quantitative NMR scanning did not reveal significant changes in fat or lean mass of Mc1r^e/e^ mice in comparison to control WT mice (Fig. 1C, D). However, chow-fed Mc1r^e/e^ mice had significantly higher gonadal (gWAT), subcutaneous (sWAT) and retroperitoneal white adipose tissue (rWAT) depot weights at sacrifice (Fig. 1E), while Western diet feeding increased particularly gWAT weight and this effect was more pronounced in Mc1r^e/e^ mice (Fig. 1F). Overall, the net weight of all WAT depots was significantly higher in chow- and Western diet-fed Mc1r^e/e^ mice compared to WT mice (Fig. 1G). Furthermore, Mc1r^e/e^ mice showed higher liver weight in both diet groups (Fig. 1H). Quantification of plasma lipids revealed a significant increase in TG concentration in Western diet fed Mc1r^e/e^ mice (Fig. 1I), while no change was observed in NEFA concentration (Fig. 1J). We next determined whether the increased adiposity of Mc1r^e/e^ mice was associated with changes in physical activity or food intake. However, no genotype effects were observed in this regard, suggesting that the increased adiposity is not primarily driven by enhanced food intake or reduced physical activity (Fig. S1A–C). We also investigated glucose homeostasis in these mice by performing glucose tolerance test (GTT). No significant differences were observed in fasting blood glucose level or glucose tolerance between WT and Mc1r^e/e^ mice (Fig, S1D–F). Additionally, plasma insulin concentration was comparable between WT and Mc1r^e/e^ mice in both diet groups (Fig. S1G). Together, these results indicate that global MC1R deficiency promotes adiposity without affecting food intake, physical activity or glucose homeostasis.

### Mc1r^e/e^ mice show enhanced adipocyte hypertrophy

**Fig. 2:**
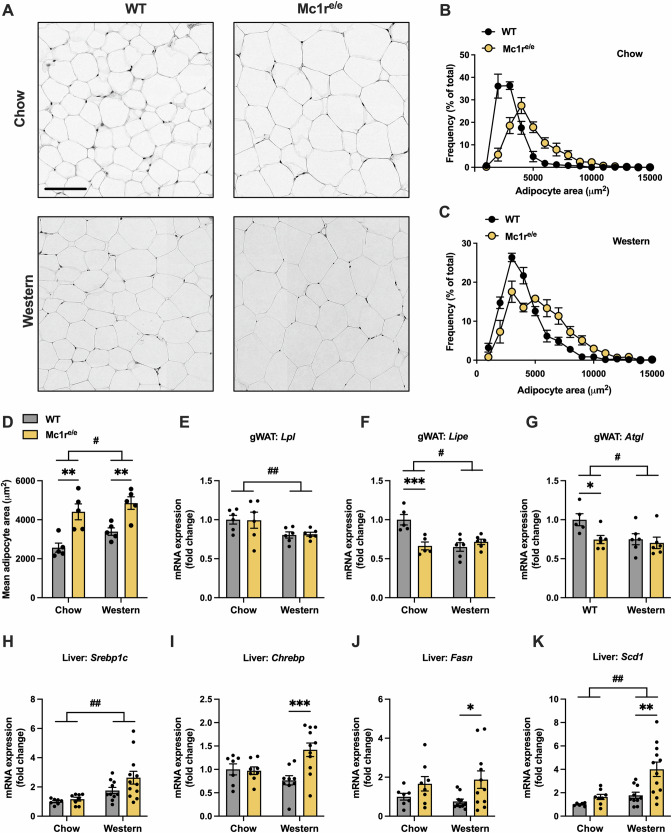
Mc1r^e/e^ mice show increased adipocyte hypertrophy and upregulation of lipogenesis-related genes in the liver. **A** Representative H&E-stained gWAT sections of chow- and Western diet-fed WT and Mc1r^e/e^ mice. Scale bar = 100 µm. **B**, **C** Adipocyte size distribution in histological gWAT samples from chow- and Western diet-fed WT and Mc1r^e/e^ mice (**D**) Mean adipocyte size in gWAT samples of chow- and Western diet-fed WT and Mc1r^e/e^ mice. **E–G** Quantitative real-time-polymerase chain reaction (qPCR) analysis of *Lpl*, *Lipe*, *Atgl* expression in the gWAT. **H–K** qPCR analysis of *Srebp1c*, *Chrebp*, *Fasn* and *Scd1* expression in the liver. Values are mean ± SEM, *n* = 10–15 mice per group in each graph. *p < 0.05, **p < 0.01 and ***p < 0.001 for the indicated comparisons by two-way ANOVA and Bonferroni post hoc tests. # *p* < 0.05 and ## *p* < 0.01 for the main effect of diet by 2-way ANOVA. gWAT indicates gonadal white adipose tissue. Lpl lipoprotein lipase, Lipe hormone sensitive lipase, Atgl adipose triglyceride lipase, Srebp1c sterol regulatory element-binding protein 1, Chrebp carbohydrate response element-binding protein, Fasn fatty acid synthase, Scd1 stearoyl-CoA desaturase 1.

We next investigated the effect of MC1R deficiency on WAT morphology. Histological examination of H&E-stained gWAT sections revealed a higher proportion of large adipocytes in both chow- and Western diet-fed Mc1r^e/e^ mice (Fig. 2A–C). Consequently, the mean adipocyte cell size was also increased in Mc1r^e/e^ mice compared to WT mice (Fig. 2D), indicating enhanced adipocyte hypertrophy. To address the possible causes of increased adipocyte hypertrophy, we first quantified mRNA levels of key enzymes involved in fatty acid hydrolysis in gWAT samples using qPCR. Although the expression of lipoprotein lipase (encoded by *Lpl*), which is a rate-limiting enzyme for the turnover of fatty acids in adipocytes [30] was unchanged (Fig. 2E), chow-fed Mc1r^e/e^ mice had significantly reduced mRNA levels of hormone sensitive lipase (HSL, encoded by *Lipe*) and adipose triglyceride lipase (*Atgl*) (Fig. 2F, G), which are two main enzymes that account for around 95% of lipolytic activity in adipocytes [30]. Meanwhile, the expression of monoglyceride lipase (*Mgl*), a rate-limiting enzyme in the final step of TG hydrolysis, lipid droplet-associated proteins (*Plin1* or *Plin2*), and genes involved in fatty acid synthesis (*Fasn, Scd1, Acc1, Dgat1, Dgat2 Adipoq, Mgll*), and fatty acid oxidation (*Ppara and Acox1*) were unchanged in gWAT of Mc1r^e/e^ mice (Fig. S2).

Earlier studies have shown that the deletion of ATGL in adipocytes reduces lipolysis and changes fat distribution without increasing overall fat mass [31], while genetic ablation of HSL protects against diet-induced and genetic obesity in mice [32, 33]. Thus, the reduced mRNA levels of *Atgl* and *Lipe* in adipocytes are unlikely to explain the expansion of fat mass in Mc1r^e/e^ mice, particularly considering that the gene expression was changed only in chow-fed Mc1r^e/e^ mice, while adipocyte hypertrophy was evident in both chow- and Western diet-fed mice. Therefore, we turned our attention to the liver and quantified mRNA expression of key enzymes involved in de novo lipogenesis in the liver of Mc1r^e/e^ mice. We did not observe any change in the mRNA level of sterol regulator element binding protein 1 (*Srebp1c*) (Fig. 2H), which primarily regulates the expression of genes involved in FA and TG biosynthesis *via* insulin, while the expression of carbohydrate response element-binding protein (*Chrebp*), another important transcription factor for de novo lipogenesis, was significantly upregulated in Western diet-fed Mc1r^e/e^ mice (Fig. 2I). Consistently, the mRNA levels of the *Chrebp* target genes fatty acid synthase (*Fasn*) and stearoyl-CoA desaturase 1 (*Scd1*) were significantly increased in the liver of both chow- and Western diet-fed Mc1r^e/e^ mice (Fig. 2J, K). Collectively, these findings suggest that the enhanced adiposity of Mc1r^e/e^ mice is primarily driven by a disturbance in hepatic de novo lipogenesis rather than adipose tissue dysfunction.

### Hepatocyte-specific loss of MC1R increases liver weight and causes dyslipidemia in mice challenged with Western diet

**Fig. 3:**
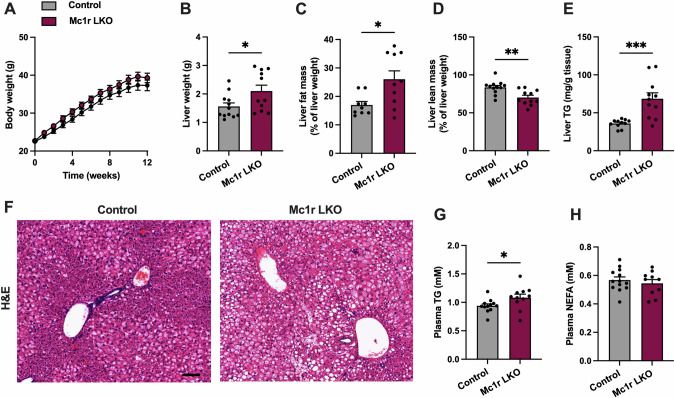
Hepatocyte-specific melanocortin 1 receptor (MC1R) deficiency increases liver weight and hepatic triglyceride accumulation. **A** Body weight curves of Western diet-fed control and Mc1r LKO mice. **B** Liver weight at the end of the experiment. **C**, **D** Quantification of fat and lean mass of whole liver by quantitative NMR scanning. **E** Quantification of liver TG levels in control and Mc1r LKO mice. **F** Representative H&E-stained liver sections of Western diet-fed control and Mc1r LKO mice. Scale bar = 100 µm. **G**, **H** Quantification of plasma TG and NEFA concentrations in control and Mc1r LKO mice. Values are mean ± SEM, *n* = 10–15 mice per group in each graph. *p < 0.05, **p < 0.01 and ***p < 0.001 by unpaired two-tailed Student’s *t* test. Mc1r LKO indicates liver-specific MC1R knockout mice; TG triglycerides, NEFA non-esterified fatty acids.

To test the hypothesis that MC1R deficiency in the liver triggers dyslipidemia and adipocyte hypertrophy, we employed hepatocyte-specific MC1R knockout (Mc1r LKO) mice, as previously described [19], fed them Western diet for 12 weeks, and then phenotyped the mouse model in a comparable way as Mc1r^e/e^ mice. Mc1r LKO displayed a similar rate of weight gain throughout the entire feeding period in comparison with control mice (Fig. 3A). Furthermore, total lean or fat mass, as determined by quantitative NMR scanning, did not significantly differ between the genotypes at the start or end of the 12-week feeding period (Fig. S3A, B). Food intake was also comparable between the genotypes (Fig. S3C). Of note, Mc1r LKO mice had increased liver weight compared to control mice (Fig. 3B), which prompted us to further investigate the relative fat and lean mass of the liver by ex vivo quantitative NMR scanning. In line with the increased liver weight, the relative fat mass of the liver was increased in Mc1r LKO mice (Fig. 3C), which was accompanied by reduction in the relative lean mass (Fig. 3D). Supporting these finding, quantification of lipids revealed increased TG concentration in the liver of Mc1r LKO mice (Fig. 3E) Histological examination of H&E-stained liver sections revealed increased accumulation of intracellular lipid droplets in Mc1r LKO mice (Fig. 3F). In terms of plasma lipid profiles, Mc1r LKO mice had increased plasma TG concentration (Fig. 3G), while no change was observed in plasma NEFA concentration (Fig. 3H). In addition, we performed GTT at the start and end of the 12-week feeding period to investigate the possible impact of hepatic MC1R deficiency on glucose homeostasis. Mc1r LKO mice showed normal glucose tolerance (Fig. S3D–G), which is in line with the finding in Mc1r^e/e^ mice. Together, these results demonstrate that hepatocyte-specific loss of MC1-R in mice increases liver weight and accumulation of TG in the liver and plasma, thus closely recapitulating the phenotype observed in Mc1r^e/e^ mice.

### Western diet-fed Mc1r LKO mice show enhanced adipocyte hypertrophy

**Fig. 4:**
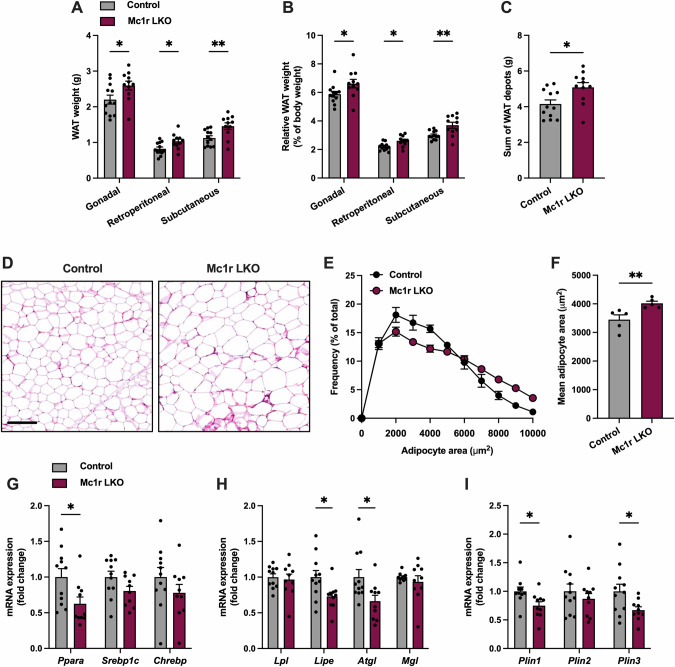
Hepatocyte-specific MC1R deficiency increases adiposity. **A** Absolute weights of different white adipose tissue (WAT) depots in Western diet-fed control and Mc1r LKO mice. **B** Relative WAT weights (expressed as % of body weight). **C** Sum weight of all WAT depots in Western diet-fed control and Mc1r LKO mice. **D** Representative H&E-stained gWAT sections of Western diet-fed control and Mc1r LKO mice. Scale bar  = 100 µm. **E**, **F** The distribution of adipocyte sizes and mean adipocyte area in the gWAT of Western diet-fed control and Mc1r LKO mice. **G–I** qPCR analysis of genes involved in lipogenesis and lipolysis, and lipid droplet-associated proteins in the gWAT of Western diet-fed control and Mc1r LKO mice. Values are mean ± SEM, *n* = 10–15 per group in each graph. *p < 0.05 and **p < 0.01 by unpaired two-tailed Student’s *t* test. Ppara peroxisome proliferator activated receptor alpha, Srebp1c sterol regulatory element-binding protein 1, Chrebp carbohydrate response element-binding protein, Lpl lipoprotein lipase, Lipe hormone sensitive lipase, Atgl adipose triglyceride lipase, Mgll monoglyceride lipase, Plin1 perilipin-1, Plin2 perilipin-2, Plin3 perilipin-3.

We next investigated whether the Mc1r LKO mouse model leads to a similar adiposity phenotype as observed in Mc1r^e/e^ mice. Indeed, Mc1r LKO mice had significantly higher absolute and relative gWAT, rWAT and sWAT depot weights at the end of the experiment (Fig. 4A, B). Consequently, net weight of all WAT depots was increased in Mc1r LKO mice (Fig. 4C). We next investigated adipose tissue morphology in H&E-stained gWAT sections from Western diet-fed control and Mc1r LKO mice. Histological examination and quantification of cross-sectional areas revealed an increase in adipocyte size in Mc1r LKO mice (Fig. 4D–F). To further explore molecular mechanisms promoting fat deposition, we quantified mRNA levels of lipid metabolism and transportation genes in the gWAT of Mc1r LKO mice. The expression of *Ppara*, which controls lipogenesis in gWAT [34], was reduced in Mc1r LKO mice, while *Srebp1c* and *Chrebp* expression were unchanged (Fig. 4G). Furthermore, the expression of genes involved in de novo lipogenesis, lipid transport and TG synthesis were comparable between the genotypes, except for *Fasn* and *Gpat3*, which were downregulated in Mc1r LKO mice (Fig. S4A). In good agreement with the gene expression profile of Mc1r^e/e^ mice, the mRNA levels of *Lipe* and *Atgl* were reduced in Mc1r LKO mice (Fig. 4E), whereas *Lpl* and *Mgll* mRNA levels were unchanged (Fig. 4E). Remarkably, the mRNA levels of the lipid droplet-associated proteins *Plin1* and *Plin3* were reduced in Mc1r LKO mice, while *Plin2* was unchanged (Fig. 4I). The expression levels of the pro-inflammatory cytokines *Il1b*, *Il6* and *Tnfa* were comparable between the genotypes (Fig. S4B). Together, these data indicate that hepatic MC1R deficiency promotes expansion of WAT depots and adipocyte hypertrophy in mice challenged with Western diet.

### Hepatic transcriptome of Mc1r LKO mice

**Fig. 5:**
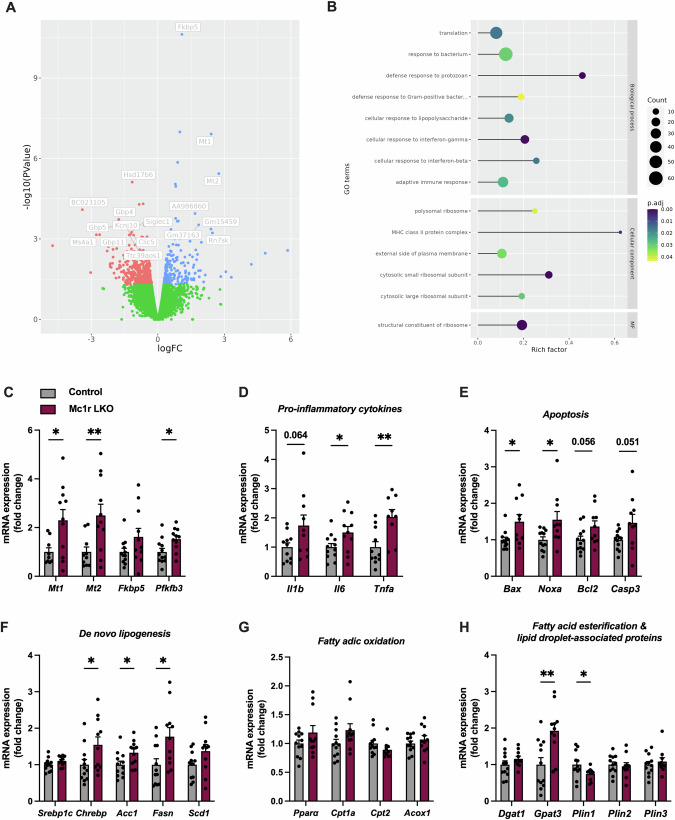
Hepatic transcriptome of Western diet-fed Mc1r LKO mice. **A** Volcano plot of differentially expressed genes (DEGs) between control and Mc1r LKO mice. *n* = 4 mice per group. **B** Gene ontology (GO) terms associated with DEGs that were identified using topGO, a Bioconductor R package. Circle size indicates the number of DEGs enriched in the pathway, and circle color indicates the degree of enrichment. **C** Validation of RNA-Seq data with qPCR of selected DEGs (*Mt1*, *Mt2*, *Fkbp5 Pfkfb3*) in the liver of Western diet-fed control and Mc1r LKO mice. **D**, **E** qPCR analysis of pro-inflammatory cytokines and apoptosis markers in the liver. **F–H** qPCR analysis of genes involved in de novo lipogenesis, fatty acid oxidation, and fatty acid esterification and lipid droplet formation. Values are ±SEM, *n* = 10–11 mice per group in each graph. *p < 0.05 versus control mice by Student’s *t* test. Mt1 metallothionein 1, Mt2 metallothionein 2, Fkbp5 FK506 binding protein 5, Il1b interleukin-1 beta, Il6 interleukin 6, Tnfa tumor necrosis factor alpha, Bax Bcl2-associated X protein, Noxa phorbol-12-myristate-13-acetate-induced protein 1, Bcl2 B-cell lymphoma 2, Casp3 caspase 3, Chrebp carbohydrate response element binding protein, Acc1 acetyl-CoA carboxylase, Fasn fatty acid synthase, Scd1 stearoyl-CoA desaturase-1, Gpat3 glycerol-3-phosphate acyltransferase 1, Ppara peroxisome proliferator-activated receptor, Cpt1a carnitine O-palmitoyltransferase 1, Cpt2 carnitine O-palmitoyltransferase 2, Acox1 peroxisomal acyl-coenzyme A oxidase 1, Dgat1 diglyceride acyltransferase, Gpat3 glycerol-3-phosphate acyltransferase 3, Plin1 perilipin-1, Plin2 perilipin-2, Plin3 perilipin-3.

Next, direct RNA sequencing was applied to identify changes in the hepatic transcriptome of Western diet-fed Mc1r LKO mice (*n* = 4) compared to Western diet-fed control mice (*n* = 4). We found 635 differentially expressed genes (DEGs) between the Mc1rLKO and control samples (adjusted *p* value < 0.05, logFC >0.25). As shown in the volcano plot (Fig. 5A), 240 genes were upregulated, and 395 genes were downregulated in Mc1r LKO mice. Furthermore, gene ontology (GO) enrichment analysis was performed on 635 DEGs to identify relevant pathways and biological processes affected by hepatic MC1R deficiency. The DEGs were significantly enriched in several GO terms including defense response to bacteria, cellular responses to lipopolysaccharide, interferon-gamma and -beta, and adaptive immune response (Fig. 5B).

We further selected a few of the top DEGs and validated these by qPCR using the whole set of liver samples from Western diet-fed control and Mc1r LKO mice. Metallothionein 1 (*Mt1*) and metallothionein 2 (*Mt2*), which are essential stress response proteins in the liver [35], were significantly upregulated in Mc1r LKO mice (Fig. 5C), thus confirming the finding of RNA-Seq analysis. Likewise, FK506 binding protein 5 (*Fkbp5*) tended (*p* = 0.10) to be upregulated and 6-phosphofructo-2-kinase (*Pfkfb3*) was significantly upregulated in the liver of Mc1r LKO mice (Fig. 5C). Based on the findings from the GO enrichment analysis, we were curious to investigate the impact of hepatic MC1R deficiency on the expression of main inflammatory genes. Mc1r LKO mice showed a tendency towards increased expression of interleukin-1 beta (*Il1b)* as well as significant upregulation of interleukin 6 (*Il6*) and tumor necrosis factor alpha (*Tnfa*), which are key pro-inflammatory cytokines regulating hepatic inflammation in NAFLD [36] (Fig. 5D). Besides inflammation, Mc1r LKO mice displayed signs of enhanced apoptosis, as evidenced by upregulation of the apoptosis markers Bcl2-associated X protein (*Bax*), phorbol-12-myristate-13-acetate-induced protein 1 (*Noxa*), B-cell lymphoma 2 (*Bcl2*, *p* = 0.056) and caspase 3 (*Casp3*, *p* = 0.051) (Fig. 5E). Histological analysis of Picrosirius Red-stained sections also showed that liver fibrosis was increased in Mc1r LKO mice (Fig S6A, B).

Although pathways related to lipid metabolism were not among the most significant GO terms in the enrichment analysis, we aimed to also quantify the hepatic expression of genes involved in lipogenesis, fatty acid oxidation, and lipid droplet formation. Consistent with the transcriptional changes in Mc1r^e/e^ mice, Mc1r LKO mice showed no change in *Srebp1c* expression but significant upregulation of *Chrebp* (Fig. 5F). Consequently, the ChREBP target genes *Acc1* and *Fasn* were also significantly induced in the liver of Mc1r LKO mice (Fig. 5F). In contrast, genes involved in fatty acid oxidation (*Ppara*, *Cpt1a*, *Cpt2* and *Acox1*), fatty acid export (*Mttp* and *Apob*) or uptake (*Cd36*) were unaffected by MC1R deficiency (Fig. 5G and Fig. S5B). In terms of lipid droplet-associated proteins, *Plin1* was downregulated Mc1r LKO mice, while *Plin2* and *Plin3* were unchanged (Fig. 5H). Furthermore, the expression of *Gpat3*, an enzyme involved in TG synthesis and catalyzing the first step of fatty acid esterification, was markedly upregulated in Mc1r LKO mice (Fig. 5H). Collectively, these expression analyses suggest that hepatic MC1R deficiency enhances de novo lipogenesis and fatty acid esterification, leading to excessive accumulation of TGs and hepatic steatosis.

### Pharmacological activation of MC1R reduces ChREBP expression in primary mouse hepatocytes

**Fig. 6:**
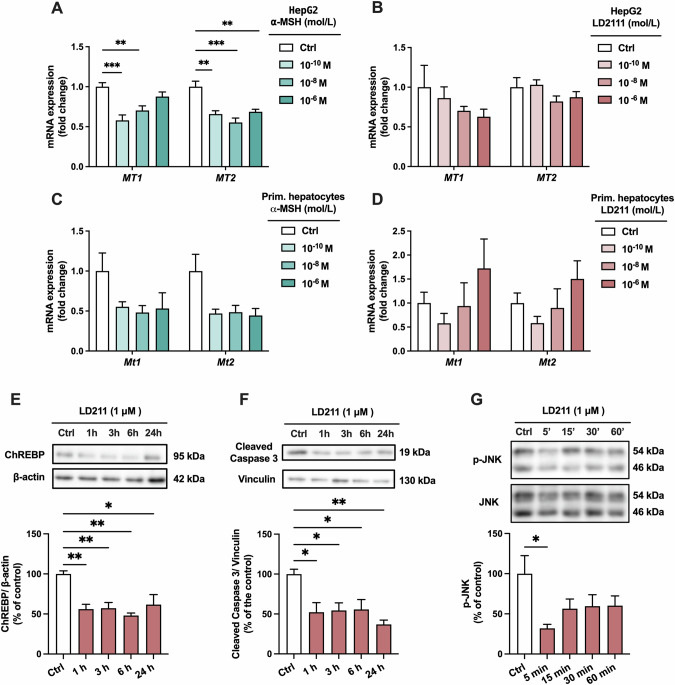
Pharmacological activation of MC1R reduces ChREBP protein expression in primary mouse hepatocytes. **A**, **B** qPCR analysis of *MT1* and *MT2* mRNA levels in HepG2 treated with different concentrations (0.1 nM, 10 nM, or 1 µM) of α-MSH or the selective MC1R agonist LD211 for 3 h. **C**, **D** qPCR analysis of *Mt1* and *Mt2* mRNA levels in primary mouse hepatocytes treated with different concentrations (0.1 nM, 10 nM, or 1 µM) of α-MSH or the selective MC1R agonist LD211 for 3 h. **E**, **F** Representative Western blots and quantification of ChREBP and cleaved caspase 3 protein levels in primary mouse hepatocytes treated with 1 µM of LD211 for 1, 3, 6 or 24 h. **G** Representative Western blots and quantification of phosphorylated JNK (p-JNK) in primary mouse hepatocytes treated with 1 µM of LD211 for 5, 15, 30 or 60 min. Values are mean ± SEM, *n* = 3–6 per group in each graph from 2 independent experiments. *p < 0.05, **p < 0.01 and ***p < 0.001 for the indicated comparisons by one-way ANOVA and Dunnet post hoc tests.

We next aimed to investigate whether pharmacological MC1R activation evokes a reverse phenotype compared to the effects observed in Mc1r LKO mice. For this purpose, we used HepG2 and primary mouse hepatocytes, and treated them with different concentrations of endogenous (α-MSH) or synthetic (LD211) MC1R agonist. In HepG2 cells, α-MSH reduced the mRNA expression of both *MT1* and *MT2* (Fig. 6A), while the selective MC1R agonist LD211 had no significant effects on the expression of these genes (Fig. 6B). Likewise, in primary hepatocytes, which represent the gold standard for investigating hepatic lipid metabolism in vitro, we observed that α-MSH tended to downregulate *Mt1* and *Mt2* expression (Fig. 6C), while LD211 had no consistent effect on the expression of these genes (Fig. 6D). We then investigated the effects of α-MSH and LD211 on ChREBP expression, which was consistently upregulated in the liver of Mc1r^e/e^ and Mc1r LKO mice. α-MSH and LD211significantly reduced ChREBP protein level in primary mouse hepatocytes (Fig. S7A, Fig. 6E). In terms of apoptosis markers, LD211 significantly reduced the protein levels of cleaved caspase 3 in primary mouse hepatocytes, while no effect on BAX was observed (Fig. S7B). Lastly, LD211 induced a rapid reduction (at 5 min time point) in the phosphorylation level of c-Jun N-terminal kinase (JNK) (Fig. 6G), which is known to mediate apoptosis in the liver [37]. Collectively, these results demonstrate that selective MC1R activation reduces the expression of ChREBP and apoptosis-related markers in primary hepatocytes.

## Discussion

In this study, we demonstrate that loss of MC1R signaling disturbs lipid metabolism in the liver and enhances adiposity. First, we found that mice with global MC1R deficiency had normal body weight and glucose homeostasis, but they were susceptible for diet-induced increase in liver weight, WAT expansion and adipocyte hypertrophy. Second, this phenotype was recapitulated in mice with hepatocyte-specific loss of MC1R. These mice, when fed Western diet, showed increased liver weight and TG accumulation in the plasma and liver as well as enhanced adiposity. At the gene expression level, these changes were associated with hepatic upregulation of the transcription factor ChREBP and its targets such as *Fasn* and *Acc1* that regulate lipogenesis. Additionally, in vitro experiments revealed that pharmacological activation of MC1R reduced ChREBP expression in primary mouse hepatocytes, indicating that ChREBP is directly regulated by MC1R signaling. These findings provide new insight into the role of melanocortin receptors in lipid metabolism.

Based on the earlier findings that MC1R is expressed in mouse and human adipocytes, we initially hypothesized that MC1R signaling in these cells regulates lipid metabolism. Mc1r^e/e^ mice with global MC1R deficiency showed normal weight development under chow-fed conditions and in diet-induced obesity, but significant increases in liver weight, plasma TG concentration and WAT depot weights. Gene expression profiling however revealed more drastic and consistent changes in the liver compared to WAT, suggesting enhanced hepatic DNL in Mc1r^e/e^ mice. Although we cannot exclude the possible involvement of adipocyte MC1R, the notion that Mc1r LKO mice displayed a highly similar phenotype with Mc1r^e/e^ mice strongly suggest that enhanced adiposity and disturbed lipid metabolism is primarily driven by MC1R deficiency in hepatocytes. This view is also supported by a previous study, which has shown that MC1R activation does not have a significant effect on lipid metabolism in cultured adipocytes [17]. Furthermore, our recent study revealed that hepatic MC1R signaling regulates cholesterol and bile acid metabolism in mice [19], indicating existence of functional MC1R in the liver.

Global and hepatocyte-specific MC1R deficiency were associated with upregulation of *Chrebp* and its target genes that regulate lipogenesis. These changes occurred without transcriptional modulation of genes regulating *e.g*. fatty acid oxidation or export, suggesting that the rate of FA input exceeds the rate of FA output, which, in turn, increases TG accumulation in the liver. Mc1r LKO mice indeed showed increased liver weight and particularly higher liver fat mass and TG content. Further studies are however needed to measure the rate of lipogenesis in these mice to confirm that the increased hepatic TG content is caused by enhanced hepatic DNL. The major transcriptional regulators SREBP1c and ChREBP induce lipogenesis in the liver by triggering the expression of key lipogenic enzymes [38]. SREBP1c and ChREBP have overlapping and yet distinct roles in lipid metabolism [39], as SREB1c is activated by insulin and ChREBP by hepatic uptake of excess plasma glucose. Intriguingly, Mc1r^e/e^ mice and Mc1r LKO mice did not show any change in fasting blood glucose level or glucose tolerance, suggesting that the ChREBP is directly induced by MC1R deficiency. In support of this view, pharmacological activation of MC1R downregulated ChREBP expression in primary mouse hepatocytes.

At first glance, it seemed that Mc1r LKO mice displayed phenotypic similarity with mice overexpressing ChREBP [40]. This mouse model has been characterized by increased hepatic expression of genes involved in lipogenesis and fatty acid esterification, and consequent increase in liver TG content and development of hepatic steatosis. However, on a high fat diet, ChREBP overexpressing mice showed improved glucose tolerance and insulin sensitivity as well as lower gonadal WAT weight, indicating that these mice are metabolically healthier than their controls. In another study, adenoviral overexpression of ChREBP led to robust hepatic steatosis that was associated with reduced blood glucose level and plasma TG concentration, and increased plasma NEFA concentration [41]. Mechanistic experiments further suggested that ChREBP overexpression, by inducing SCD1 expression, favorably modifies lipid composition in hepatocytes and thus dissociates hepatic steatosis from insulin resistance [40]. Since Western diet-fed Mc1r^e/e^ and Mc1r LKO mice showed a discordant phenotype in this regard, i.e. significantly increased WAT weights and plasma TG level without improvement in glucose tolerance, the upregulation of *Chrebp* alone could not be responsible for the increased adiposity of MC1R deficient mice. Therefore, MC1R deficiency in hepatocytes is likely to disturb additional, as yet undiscovered pathways that regulate whole body lipid metabolism.

A striking observation of MC1R deficient mouse models was increased adiposity and adipocyte hypertrophy without change in body weight. Adipose tissue has capacity to expand in size to store excess energy in the form of TGs [42]. Thus, it is likely that MC1R deficient mice were over time increasing adipocyte size and WAT mass as a rescuing mechanism to compensate for the increased lipogenesis in the liver. Interestingly, the mRNA levels of *Lipe* and *Atgl*, which are responsible for TG hydrolysis and facilitate FA mobilization, were reduced in the adipose tissue of Mc1r^e/e^ mice and Mc1r LKO mice. Likewise, perilipins and particularly *Plin1*, which is required for maximal lipolytic activity in WAT [43, 44], was downregulated in Mc1r LKO mice. Taking into account that Western diet feeding similarly downregulated *Lipe*, *Atgl* and *Plin1* expressions in WT mice, these transcriptional changes could reflect a compensatory mechanism to suppress lipolysis and FA flux to the liver. However, plasma NEFA level, which is largely determined by the rate of lipolysis, was unchanged in MC1R deficient mice, suggesting that lipolysis in the adipose tissue was still under homeostatic balance or only marginally reduced. Importantly, the unchanged plasma NEFA level support the concept that enhanced hepatic DNL is the primary mechanism driving hepatic TG accumulation in Mc1r LKO mice. We also observed that *Pppara* was downregulated in the WAT of Mc1r LKO mice, which could indicate reduced FA oxidation as an adaptive mechanism to accommodate storage of excess TGs fluxed from the liver. The expansion of WAT is an adaptive process to store excess energy, but in the context of sustained obesity, the adaptive mechanisms eventually fail leading to WAT dysfunction characterized by insulin resistance, inflammation and fibrosis [45]. Insulin resistance in WAT is particularly important as a causative factor for hepatic TG accumulation in NAFLD [46]. Based on the unchanged plasma NEFA level and gene expression analysis, there were no signs of insulin resistance or increased inflammation in the WAT of Mc1r LKO mice, implying that the capacity of the WAT to safely store excess FAs was not yet exceeded. It remains however an open question whether feeding a Western diet for a longer period of time or a diet with higher fat percentage would lead to WAT dysfunction in Mc1r LKO mice. RNA-Seq analysis revealed that several biological processes are affected by hepatocyte-specific MC1R deficiency. In terms of the top DEGs, Mc1r LKO mice showed significant upregulation of the metallothioneins *Mt1* and *Mt2*, which are cysteine-rich heavy metal-binding proteins that are expressed in various tissues with particularly high expression levels in the liver. They are induced by various stimuli such as heavy metals, oxidate stress and inflammation, and are thus implicated e.g. in scavenging of free radicals and protection against heavy metal toxicity. Mice with targeted disruption of *Mt1* and *Mt2* are also more prone to diet-induced obesity, indicating a role for metallothioneins in the regulation of energy balance [47, 48]. Furthermore, overexpression of metallothioneins protects hepatocytes against palmitic acid-induced lipotoxicity [49]. Against this background, it is more likely that the upregulation of *Mt1* and *Mt2* in the liver of Western diet-fed Mc1r LKO mice was a response to enhanced TG accumulation rather than a direct consequence of MC1R deficiency. This notion is further supported by the finding that selective MC1R agonism did not affect *Mt1* or *Mt2* expression in cultured hepatocytes.

Besides susceptibility for increased hepatic TG accumulation, Western diet-fed Mc1r LKO mice exhibited signs of enhanced liver fibrosis and inflammation as well as apoptosis. We had previously observed that selective activation of MC1R had no effect on inflammatory markers but reduced the expression of fibrosis-associated genes in cultured hepatocytes [19]. In the current study, we extend this knowledge by showing that MC1R activation has some direct effects on apoptotic markers in primary hepatocytes. Overall, the in vitro data suggest that hepatocyte-specific loss of MC1R could directly promote liver fibrosis and apoptosis, but the pathological hallmarks of NAFLD, *i.e*. apoptosis, inflammation and fibrosis, might also be a consequence of increased TG accumulation in the liver. Although TG accumulation is not considered to be harmful for hepatocytes [50], lipotoxicity might have occurred in Mc1r LKO mice if the capacity of the liver to store and export NEFAs as TGs was exceeded due to increased DNL. In particular, saturated NEFAs as well as free cholesterol are mediators of lipotoxicity by activating apoptotic signaling pathways [51]. Apoptotic cells are then cleared by Kupffer cells, which release pro-inflammatory cytokines and activate hepatic stellate cells to promote fibrosis [52].

Overall, the present findings highlight that MC1R deficiency in the liver disturbs lipid metabolism and increases susceptibility for diet-induced adiposity without affecting body weight or glucose homeostasis. Thus, it appears that MC1R signaling in the liver complements the other well-established roles of MCR subtypes in the regulation of energy and glucose homeostasis. For instance, MC4R deficiency causes severe hyperphagia, obesity and insulin resistance in mice and humans [53, 54]. Furthermore, melanocortins regulate glucose homeostasis *via* multiple pathways: e.g. by modulating insulin secretion *via* MC4R in the CNS and by modulating glucose uptake in the skeletal muscle *via* MC5R [55, 56]. In contrast, MC1R deficiency in the liver does not lead to obesity or diabetes, but it likely enhances DNL and promotes fibrosis, apoptosis and inflammation, which in turn, predisposes to the development of NAFLD.

In conclusion, the present study identifies MC1R as an important regulator of lipid metabolism in the liver. Hepatocyte-specific loss of MC1R upregulated the expression of lipogenesis-related genes, enhanced hepatic TG accumulation and led to an adiposity phenotype characterized by increased adipocyte hypertrophy and WAT mass. MC1R deficiency also promoted signs of liver fibrosis, inflammation and apoptosis, which are pathological hallmarks of progression from simple steatosis to steatohepatitis.

## Supplementary information

Supplementary Information

## Data Availability

The raw sequence reads (FASTQ files) have been deposited in the Gene Expression Omnibus (GEO) of National Center for Biotechnology Information (NCBI) under accession no. GSE262618.
