# Supplementary material for: Hepatocyte-specific loss of melanocortin 1 receptor disturbs fatty acid metabolism and promotes adipocyte hypertrophy
Source: Int J Obes (Lond). 2024 Aug 8;48(11):1625–37. doi: 10.1038/s41366-024-01600-9 (PMC11502480; doi:10.1038/s41366-024-01600-9)
Supplement: Supplementary file 1 — Supplementary Information [file 41366_2024_1600_MOESM1_ESM.pdf]

SUPPLEMENTARY INFORMATION

Hepatocyte-specific loss of melanocortin 1 receptor disturbs fatty acid metabolism and promotes adipocyte hypertrophy

Keshav Thapa [1,2], Bishwa Ghimire [3,4], Kisun Pokharel [5], Minying Cai [6], Eriika Savontaus [1,7,8], Petteri Rinne* [1, 7]

[1] Research Centre for Integrative Physiology and Pharmacology, Institute of Biomedicine, University of Turku, Turku, Finland

[2] Drug Research Doctoral Programme (DRDP), University of Turku, Turku, Finland.

[3] Institute for Molecular Medicine Finland (FIMM), HiLIFE Helsinki Institute of Life Science, University of Helsinki, Helsinki, Finland

[4] Faculty of Medicine, University of Turku, Turku, Finland

[5] Natural Resources Institute Finland (Luke), Jokioinen, Finland

[6] Department of Chemistry and Biochemistry, University of Arizona, Tucson, Arizona, USA

[7] Turku Center for Disease Modeling, University of Turku, Turku, Finland

[8] Unit of Clinical Pharmacology, Turku University Hospital, Turku, Finland

* Corresponding author: Petteri Rinne, PhD

Institute of Biomedicine, University of Turku, Kiinamyllynkatu 10, 20520 Turku, Finland
Phone: +358-505609497, E-mail: pperin@utu.fi

Table S1. Quantitative RT-PCR primers for mouse genes.

**Gene name**<br>Accession number	**5'-3' primer sequence**
Acc1<br>NM_133360.3	Forward: gcgtcgggtagatccagtt<br>Reverse: ctcagtggggcttagctctg
Acox1<br>NM_015729.4	Forward: gcccaactgtgacttccatc<br>Reverse: gccaggactatcgcatgatt
Adipoq<br>NM_0.009605.5	Forward: ggagagaaaggagatgcaggt<br>Reverse: ctttcctgccaggggttc
Apob<br>NM_009693.2	Forward: acagtgtgcaaggatctgga<br>Reverse: agacatctgtggtcccaagg
Atgl (Pnpla2)<br>NM_025802.3	Forward: caacgccactcacatctacgg<br>Reverse: ggacacctcaataatgttggcac
Bax<br>NM_0007527.4	Forward: aaactggtgctcaaggccc<br>Reverse: cttggatccagacaagcagc
Bcl2<br>NM_009741.5	Forward: atgcctttgtggaactatatggc<br>Reverse: ggtatgcacccagagtgatgc
Casp3<br>NM_009810.3	Forward: tggtgatgaaggggtcatttatg<br>Reverse: ttcggctttccagtcagactc
Cd36<br>NM_001159558.1	Forward: ccaagctattgcgacatgatt<br>Reverse: tctcaatgtccgagacttttca
Chrebp<br>NM_021455.5	Forward: cagcccagcctagatgactt<br>Reverse: caaagctgggggactctatg
Cpt1a<br>NM_013495.2	Forward: gctgtcaaagataccgtgagc<br>Reverse: tctccctccttcatcagtgg
Cpt2<br>NM_009949.2	Forward: cagcacagcatcgtaccca<br>Reverse: tcccaatgccgttctcaaaat
Crat<br>NM_007760.4	Forward: gccattgctatgcacttcaac<br>Reverse: ggtccgaagaacatgacaca
Dgat1<br>NM_010046.4	Forward: gccacaatcatctgcttccc<br>Reverse: ccactgaccttcttccctgt
Dgat2<br>NM_026384.3	Forward: ccaagaaaggtggcagga<br>Reverse: tgaagttacagaaggcaccc
Fabp4<br>NM_024406.4	Forward: ggatggaaagtcgaccacaa<br>Reverse: tggaagtcacgcctttcata
Fasn<br>NM_007988.3	Forward: gctgctgttggaagtcagc<br>Reverse: agtgttcgttcctcggagtg

Gene name<br>Accession number	5’-3’ primer sequence
Fkbp5<br>NM_010220.4	Forward: ctagtgacgaggccccaatg<br>Reverse: cagccttccaggtggactt
Gpat3<br>NM_172715.3	Forward: ggccttcggattatccctgg<br>Reverse: cttgggggctcctttctgaa
Il1b<br>NM_008361.4	Forward: tgtaatgaaagacggcacacc<br>Reverse: tcttctttgggtattgcttgg
Il6<br>NM_031168.2	Forward: acaaccacggccttccctactt<br>Reverse: cacgatttcccagagaacatgtg
Lipe<br>NM_0010719.5	Forward: gcgctggaggagtgttttt<br>Reverse: cgctctccagttgaaccaag
Lpl<br>NM_008509.2	Forward: ctcgctctcagatgccctac<br>Reverse: aggcctggttgtgttgctt
Mgl<br>NM_001166251.2	Forward: cggacttccaagtttttgtcaga<br>Reverse: gcagccactaggatggagat
Mt1<br>NM_013602.3	Forward: accttctcctcacttactccgtagc<br>Reverse: gctgggttggtccgatactatttac
Mt2<br>NM_008630.2	Forward: gcctgcaaatgcaaacaatgc<br>Reverse: agctgcacttgtcggaagc
Mttp<br>NM_008642.3	Forward: tcacacaactggcctctcat<br>Reverse: tgacccgcattttcaacgtt
Noxa (Pmaip1)<br>NM_021451.2	Forward: gcagagctaccacctgagttc<br>Reverse: ctttgcgacttcccaggca
Pfkfb3<br>NM_001177752.1	Forward: caactccccaaccgtgattgt<br>Reverse: gaggtagcgagtcagcttctt
Plin1<br>NM_001113471	Forward: gagaggagacagacgacgag<br>Reverse: ggtgttccggagagtgttc
Plin2<br>NM_007408.3	Forward: gttttggggatggtgcagtt<br>Reverse: ccagccgttcatagttgctc
Plin3<br>NM_025836.3	Forward: cagctaacaagtgtgctcgg<br>Reverse: tgtccctgaacatgctgagt
Ppara<br>NM_011144.6	Forward: cacgcatgtgaaggctgtaa<br>Reverse: gctccgatcacacttgtcg
Ppia<br>NM_008907.2	Forward: ccctggcacatgaatcctgg<br>Reverse: gagctgtttgcagacaaagttc
S18A (Mrps18a)<br>NM_026768.3	Forward: cagctccaagcgttcctgg<br>Reverse: ggccttcaattacagtcgtct
Scd1<br>NM_009127.4	Forward: cattctcatggtcctgctgc<br>Reverse: tgccttgtaagttctgtggc

**Gene name**<br>Accession number	**5’-3’ primer sequence**
Srebp1c<br>NM_001358314.1	Forward: gatgtgcgaactggacacag<br>Reverse: catagggggcgtcaaacag
Tnfa<br>NM_013693.1	Forward: ctgaacttcggggtgatcgg<br>Reverse: ggcttgtcactcgaattttgaga

Table S2. Quantitative RT-PCR primers for human genes.

**Gene name**<br>Accession number	**5'-3' primer sequence**
GAPDH<br>NM_002046.7	Forward: tcaaggctgagaacgggaag<br>Reverse: cgccccacttgattttggag
MT1<br>NM_005946.3	Forward: cttgggatctccaacctcac<br>Reverse: aggagcagcagctcttcttg
MT2<br>NM_0005953.5	Forward: atcccaactgctcctgcgccg<br>Reverse: cagcagctgcacttgtccgacg
RPS18<br>NM_022551.3	Forward: cgccgctagaggtgaaattc<br>Reverse: ccagtcggcatcgtttatgg

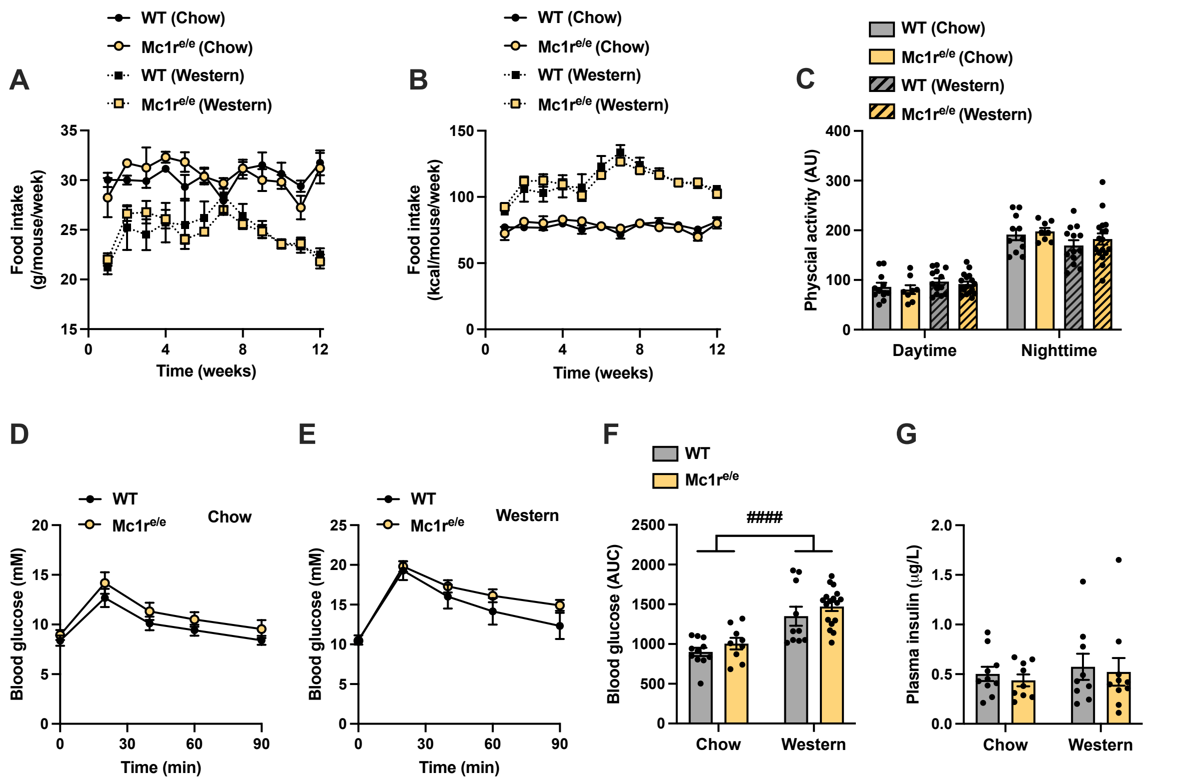

Fig. S1. Global MC1-R deficiency does not affect food intake, physical activity or blood glucose levels. (**A** and **B**) Food intake, expressed as g/week and kcal/week, in chow and Western diet-fed WT and Mc1r$^{e/e}$ mice. n=3 cages/group from 11 (WT, Chow), 9 (Mc1r$^{e/e}$, Western), 10 (WT, Western) and 12 (Mc1r$^{e/e}$, Western). Food intake per mouse was estimated from the overall consumption of group-housed mice. (**C**) Physical activity during daytime and nighttime. (**D** and **E**) Glucose tolerance test (GTT) performed at the end of the 12-week diet intervention in chow- and Western diet-fed WT and Mc1r$^{e/e}$ mice. For GTT, mice were fasted for 4 hours and then intraperitoneally injected with glucose at a dose of 2.5 g/kg lean mass. (**F**) Area under the blood glucose curves. (**G**) Plasma insulin level in chow- and Western diet-fed WT and Mc1r$^{e/e}$ mice. Values are mean ± SEM, mice n = 9-17 mice per group in each graph. #### $p<0.0001$ for the main effect of diet by 2-way ANOVA.

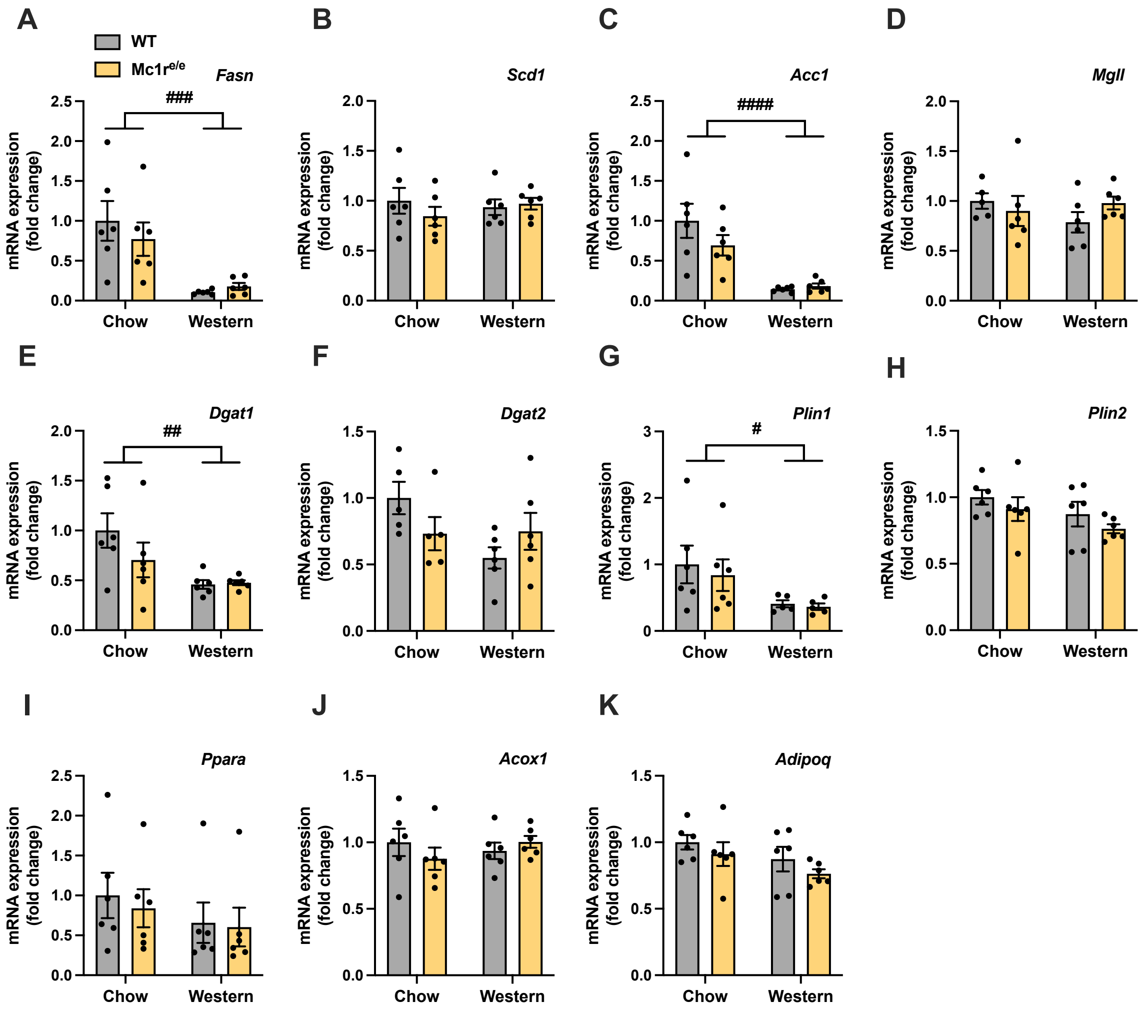

Fig. S2. Gene expression profile in gonadal white adipose tissue (gWAT) of Mc1r$^{e/e}$ mice. qPCR analysis of genes responsible for lipogenesis (**A-C**), lipolysis (**D**), fatty acid esterification (**E** and **F**), lipid droplet-associated proteins (**G** and **H**) and fatty acid oxidation (**I-K**) in the gWAT of chow and Western diet-fed WT and Mc1r$^{e/e}$ mice. Values are mean ± SEM, mice n = 6 mice per group in each graph. # p<0.01, ## p<0.01, ### p<0.001 and #### p<0.0001 for the main effect of diet by 2-way ANOVA. *Fasn,* fatty acid synthase; *Scd1,* stearoyl-CoA desaturase 1; *Acc1,* acetyl-CoA carboxylase 1, *Mgll*, monoglyceride lipase; *Dgat1,* diacylglycerol O-acyltransferase 1; *Dgat2,* diacylglycerol O-acyltransferase 2; *Plin1,* perilipin 1; *Plin2,* perilipin 2; *Ppara,* peroxisome proliferator activated receptor alpha; *Acox1,* acyl-CoA oxidase 1; *Adipoq,* adiponectin.

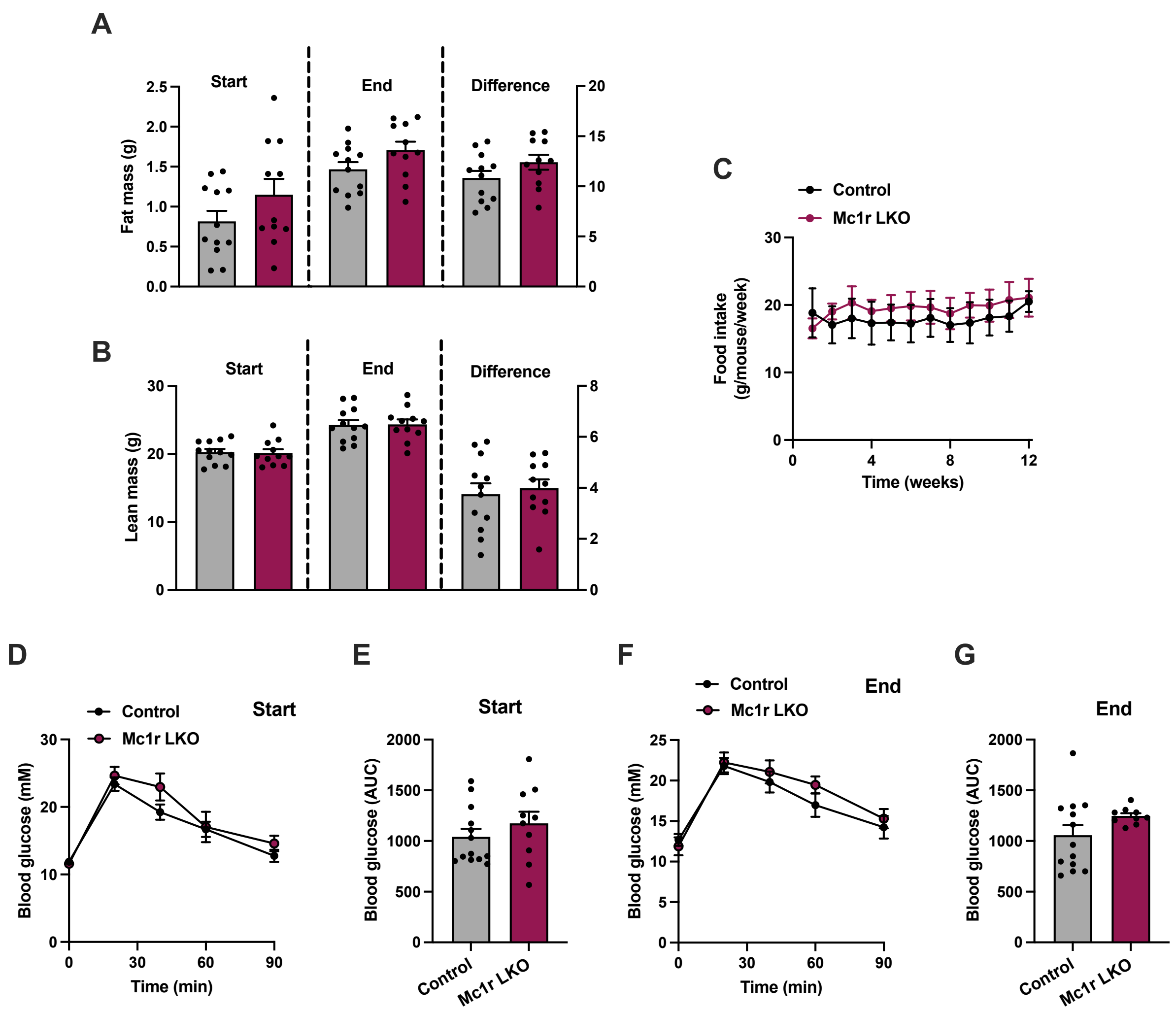

Fig. S3. Hepatocyte-specific MC1R deficiency does not affect food intake or blood glucose levels in Western diet-fed mice. (**A** and **B**) Total fat and lean mass of Mc1r LKO and control mice at the start and end of the 12-week diet intervention. The change in fat and lean mass from the start to the end of the experiment is also shown in graphs. (**C**) Food intake, expressed as g/week, in Western diet-fed Control and Mc1r LKO mice. n=3 cages/group from 7 (Control) and 6 (Mc1r LKO) mice. Food intake per mouse was estimated from the overall consumption of group-housed mice. Cages with mixed genotypes could not be used for the estimation of food intake and thus the number of mice is lower than in other analyses. (**D** and **F**) Glucose tolerance test performed at the start and end of the 12-week diet intervention in Control and Mc1r LKO mice. For GTT, mice were fasted for 4 hours and then intraperitoneally injected with glucose at dose of 2.5 g/kg lean mass. (**E** and **G**) Areas under the blood glucose curves. Values are mean ± SEM, mice n = 12 mice in Control, n = 11 mice in Mc1r LKO.

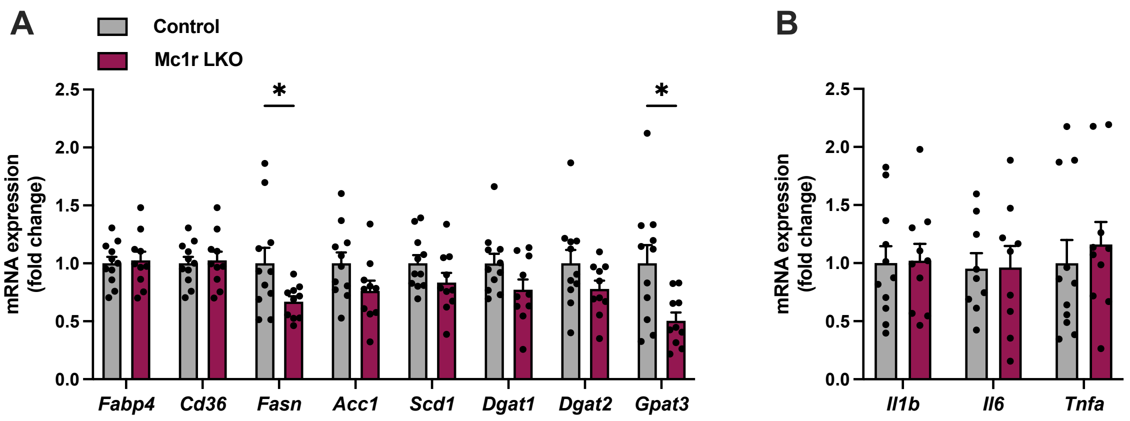

Fig. S4. Hepatocyte-specific MC1R deficiency does not alter the expression of inflammatory markers in the white adipose tissue. qPCR analysis of lipid metabolism-related genes (**A**) and pro-inflammatory cytokines (**B**) in the gWAT of Western diet-fed Control and Mc1r LKO mice. Values are mean ± SEM, mice n = 11-12 mice per group in each graph. *p<0.05 versus Control mice by unpaired Student's t test. *Fabp4,* fatty acid binding protein 4; *CD36,* cluster of differentiation 36; *Fasn,* fatty acid synthase; *Acc1*, acetyl-CoA carboxylase; *Scd1,* stearoyl-CoA desaturase-1; *Dgat1,* diglyceride acyltransferase 1, *Dgat2,* diglyceride acyltransferase 2; *Gpat3*, glycerol-3-phosphate acyltransferase 3; *Il1b,* interleukin 1 beta; *Il6,* interleukin 6; *Tnf,* tumor necrosis factor.

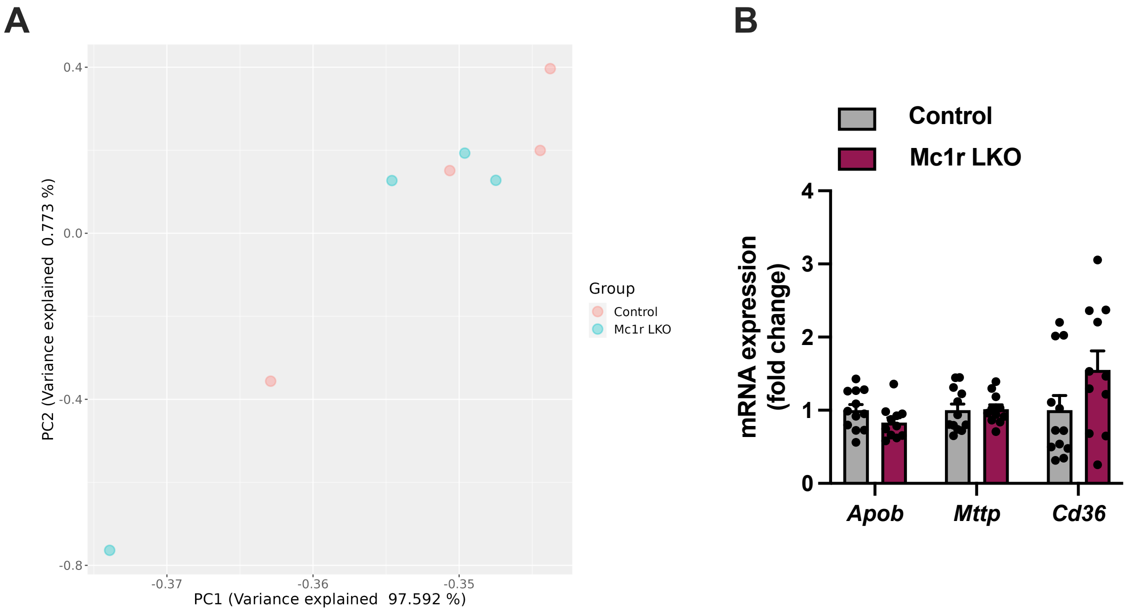

Fig. S5. Hepatic transcriptome in Mc1r LKO mice. (**A**) Principal component analysis (PCA) for the RNA-seq data from Western diet-fed Control and Mc1r LKO mice. The percentage value in the bracket represents the percentage of variance explained by that principal component. n = 4 mice per group. (**B**) qPCR analysis of genes involved in fatty acid export (*Apob*, *Mttp*) and uptake (*Cd36*) in the liver of Western diet-fed Control and Mc1r LKO mice. Values are mean ± SEM, mice n = 11-12 mice per group. *Apob*, apolipoprotein B; *Mttp*, microsomal triglyceride transfer protein; *Cd36,* cluster of differentiation 36.

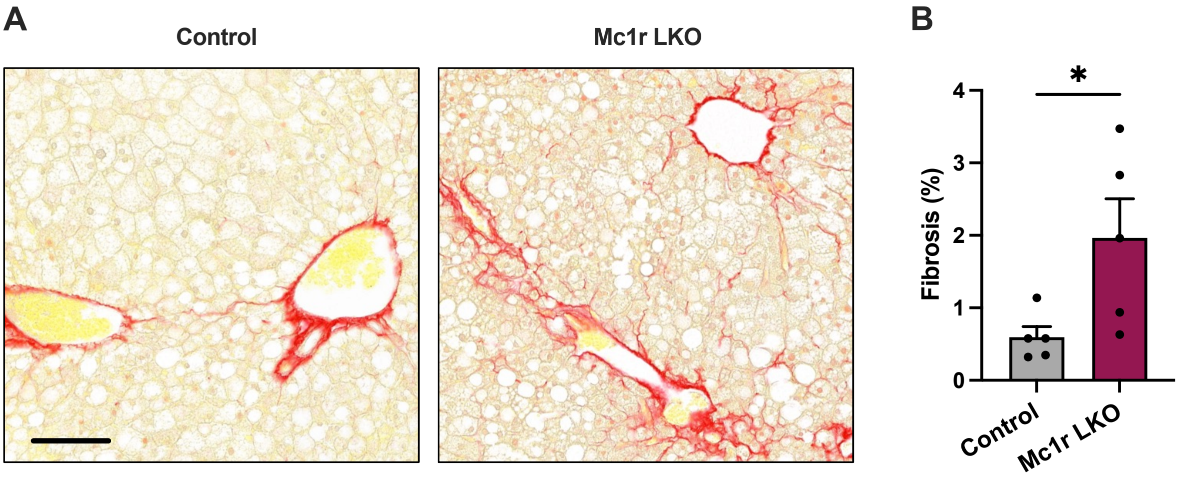

Fig. S6. Western diet-fed Mc1r LKO mice show enhanced liver fibrosis. (**A**) Representative Picrosirius Red-stained liver sections of Western diet-fed Control and Mc1r LKO mice. (**B**) Quantification of liver fibrosis as percentage of section area. Values are mean ± SEM, n = 5 mice per group. *p<0.05 versus Control mice by unpaired Student's t test.

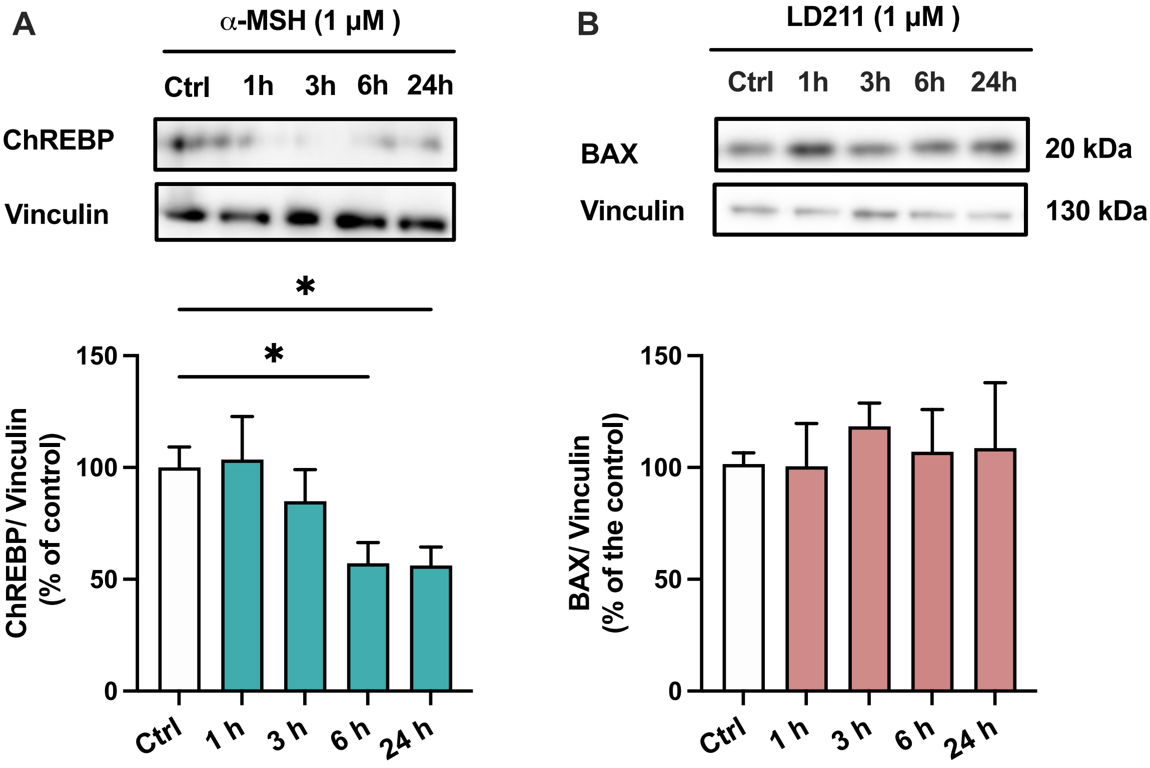

Fig. S7. α-MSH. (**A**) Representative Western blots and quantification of ChREBP protein level (normalized against vinculin) in primary mouse hepatocytes treated with 1 µM α-MSH for 1, 3, 6, or 24 hr. (**B**) Representative Western blots and quantification of BAX protein level (normalized against vinculin) in primary mouse hepatocytes treated with 1 µM α-MSH for 1, 3, 6, or 24 hr. Values are mean ± SEM, n = 3-4 per group. *$p<0.05$ for the indicated comparisons by 1-way ANOVA and Dunnet *post hoc* tests.
